# Atomistic mechanisms of water vapor–induced surface passivation

**DOI:** 10.1126/sciadv.adh5565

**Published:** 2023-11-01

**Authors:** Xiaobo Chen, Weitao Shan, Dongxiang Wu, Shyam Bharatkumar Patel, Na Cai, Chaoran Li, Shuonan Ye, Zhao Liu, Sooyeon Hwang, Dmitri N. Zakharov, Jorge Anibal Boscoboinik, Guofeng Wang, Guangwen Zhou

**Affiliations:** ^1^ Materials Science and Engineering Program and Department of Mechanical Engineering, State University of New York at Binghamton, Binghamton, NY 13902, USA.; ^2^ Department of Mechanical Engineering and Materials Science, University of Pittsburgh, Pittsburgh, PA 15261, USA.; ^3^ Department of Electrical and Computer Engineering, State University of New York at Binghamton, Binghamton, NY 13902, USA.; ^4^ Center for Functional Nanomaterials, Brookhaven National Laboratory, Upton, NY 11973, USA.

## Abstract

The microscopic mechanisms underpinning the spontaneous surface passivation of metals from ubiquitous water have remained largely elusive. Here, using in situ environmental electron microscopy to atomically monitor the reaction dynamics between aluminum surfaces and water vapor, we provide direct experimental evidence that the surface passivation results in a bilayer oxide film consisting of a crystalline-like Al(OH)_3_ top layer and an inner layer of amorphous Al_2_O_3_. The Al(OH)_3_ layer maintains a constant thickness of ~5.0 Å, while the inner Al_2_O_3_ layer grows at the Al_2_O_3_/Al interface to a limiting thickness. On the basis of experimental data and atomistic modeling, we show the tunability of the dissociation pathways of H_2_O molecules with the Al, Al_2_O_3_, and Al(OH)_3_ surface terminations. The fundamental insights may have practical significance for the design of materials and reactions for two seemingly disparate but fundamentally related disciplines of surface passivation and catalytic H_2_ production from water.

## Teaser

Real-time electron microscopy observations reveal H_2_O-induced Al(OH)_3_/Al_2_O_3_ bilayer oxide film growth on aluminum.

## INTRODUCTION

The interaction of water with solid surfaces is central to many surface chemical processes such as geochemistry, corrosion, catalysis, and electrochemistry (*1*–*5*). Many studies exist that detail the behavior of water across small lengths and time scales by carefully dosing small amounts of water onto a solid surface at cryogenic temperatures (*6*, *7*). While this approach has been successful in revealing the structure properties within the adlayer of condensed water, the resulting understanding obtained under such rarefied conditions does not translate into an equally good understanding of surface phenomena occurring in technologically relevant conditions, where the chemical reactions between adsorbed water and the solid become highly relevant. The development of aberration-corrected environmental transmission electron microscopy (ETEM) opens a unique window of atomically understanding gas-surface reactions under realistic conditions of pressure and temperature. This is exemplified by the ETEM observations of the various surface oxidation phenomena of aluminum (Al) in dry O_2_, showing the liquid-like self-healing of the amorphous Al oxide film to match the deformation of the Al substrate (*8*), crystal orientation–dependent Al oxide film nucleation and growth (*9*), and a two-stage oxidation process starting from intralayer atomic disordering to interlayer disordering leading to the formation of an amorphous Al oxide layer (*10*).

Here, we use ETEM to directly probe water vapor–induced oxide film growth on Al surfaces. Specifically, we illustrate how water vapor induces dynamic transformations of the metal lattice into its oxides at room temperature, which has not been attained yet at the atomic scale but is practically important because of the wide use of Al for applications where corrosion resistance is required (*8*, *11*–*15*). On the basis of directly observed Al(OH)_3_/Al_2_O_3_ bilayer film growth consisting of an upper layer of crystalline-like Al(OH)_3_ and an inner layer of amorphous Al_2_O_3_ on Al surfaces in water vapor, this work demonstrates the tunability of the H_2_O dissociation pathway at the ambient temperature. That is, H_2_O molecules dissociate into OH and H on pristine Al and amorphous Al_2_O_3_ to result in the surface hydrolysis into Al(OH)_3_. The Al(OH)_3_ layer facilitates the dissociation of H_2_O molecules into H_2_ molecules due to the inward O diffusion for the Al_2_O_3_ interfacial growth to a limiting thickness. These results not only offer the microscopic mechanism underlying the onset of a surface passivation process and its subsequent progression toward the self-limiting regime but also demonstrate the tunability of the dissociation pathways of H_2_O molecules with the hydroxylation of Al surfaces. These fundamental insights may have practical implications, related not only to the microscopic processes of the passivating film growth but also to catalytic H_2_ production from H_2_O over the spontaneously formed Al hydroxide overlayer on Al.

## RESULTS

### Al(OH)_3_/Al_2_O_3_ bilayer film growth on Al surfaces in H_2_O vapor

**Fig. 1.**
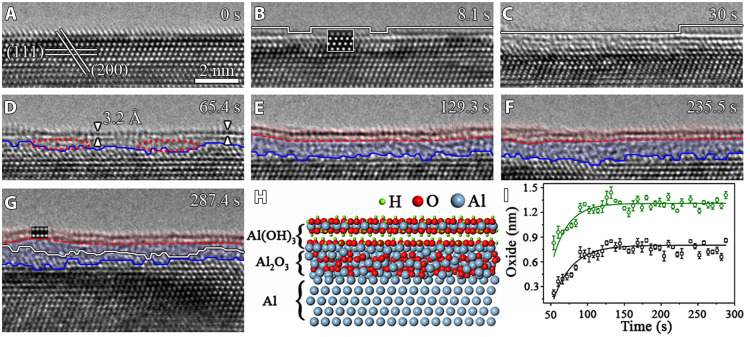
Water vapor–induced surface passivation of pristine Al(111). (**A** to **G**) Time-sequence high-resolution TEM (HRTEM) images (movie S1) showing the Al(OH)_3_/Al_2_O_3_ bilayer film growth at 298 K in pH_2_O ≈ 3.5 × 10^−5^ torr. The solid white lines highlight the weakened lattice contrast regions owing to the H_2_O adsorption–induced extraction of Al atoms from the outermost surface layer of pristine Al(111). The dashed red lines and solid blue lines mark the Al(OH)_3_/Al_2_O_3_ and Al_2_O_3_/Al(111) interfaces, respectively. The solid white line in (G) is the superimposed trace of the position and profile of the Al_2_O_3_/Al(111) interface at *t* = 65.4 s in (D). The insets in (B) and (G) are simulated HRTEM images based on the Al lattice with atomic vacancies in the topmost layer and the Al(OH)_3_ structure, respectively. (**H**) Schematic atomic view of the Al(OH)_3_/Al_2_O_3_ bilayer structure. (**I**) Time dependence of the Al(OH)_3_/Al_2_O_3_ bilayer film thickness (green) and the Al_2_O_3_/Al(111) interface displacement distance (black), where the average thickness of the oxide across the whole surface (within the field of view) is measured at ~60 s of the H_2_O dosing, after which the surface is fully covered by the Al(OH)_3_ layer that stays at a constant thickness of ~5.0 Å, while the inner amorphous Al_2_O_3_ layer grows to a limiting thickness. The error bars represent SD uncertainties based on multiple measurements on the in situ TEM images.

Figure 1 illustrates in situ high-resolution TEM (HRTEM) images, in cross-sectional view along the $\left[1\bar{1}0\right]$ zone axis, revealing the dynamic transformation of Al lattice into Al oxides while exposing a clean Al(111) surface at 298 K to 3.5 × 10^−5^ torr of water vapor. As seen in Fig. 1A, the interplanar spacing of 2.3 Å corresponds to the Al(111) planes (more details in the sections "Sample preparation" and "In situ HRTEM experiments and fig. S1). The shape of the atom columns in the topmost layer surface shows some elongation along the (200) lattice planes, which may originate from the lattice damage induced by the condensed electron beam bombardment inside the TEM to sputter off air-formed native oxide. Because the native oxide on Al is amorphous, the continuation of the crystal lattice planes along the (200) lattice plane to the topmost layer suggests that the as-prepared Al(111) surface is largely oxide-free. Upon the H_2_O exposure, some areas of the outermost layer show weakened image contrast (marked by solid white lines in Fig. 1 (B and C) due to the formation of atomic vacancies in the topmost layer, as shown by the simulated HRTEM image (Fig. 1B, inset). This indicates the extraction of Al atoms from the topmost layer as a result of the large reaction exothermicity of dissociative adsorption of H_2_O molecules. This is also consistent with many surface science experiments and the prediction from atomistic simulations, showing that the dissociative adsorption of gas molecules on metallic surfaces typically results in one–atomic layer–deep pits and adatoms extracted from the surface (*16*–*21*). The exfoliation of the outermost Al results in the hydroxylation of the two inner atomic layers, which leads to the expansion of the interplanar spacing from 2.3 Å of the pristine Al(111) lattice (Fig. 1A) to ~3.2 Å (distance between the center of the atom columns in the two topmost layers) in the region highlighted by the triangular markers in Fig. 1D, the latter of which matches well the interplanar spacing of Al(OH)_3_(112) planes (*22*–*24*).

Continued H_2_O exposure results in the attack toward deeper atomic layers of the Al substrate, as shown by the loss of lattice contrast in local regions marked with dashed red circles in Fig. 1D. Upon further H_2_O exposure, the crystalline Al lattice in the subsurface region gradually transforms into amorphous, Al_2_O_3_-like oxide, as seen from the time-sequence images in Fig. 1 (E to G). By contrast, the Al(OH)_3_ overlayer maintains the crystalline state with clearly visible image contrast of individual atom columns, as further evidenced by the inset HRTEM simulation image. The in situ TEM observation indicates that the H_2_O exposure results in the Al(OH)_3_/Al_2_O_3_ bilayer film growth, which is also cross-validated by x-ray photoelectron spectroscopy (XPS) measurements of the surface chemistry shown later. The Al_2_O_3_/Al(111) interface is atomically rough and displays an overall inward movement toward the Al side. This is evident from the detailed tracing of the movement of the Al_2_O_3_/Al(111) interface depicted in Fig. 1G, where the relative positions of the interface at 65.4 and 287.4 s are given for comparison and show that the interface moves toward the metal side by ~3.5± 0.5 Å within an elapsed time of 222 s. By contrast, the upper Al(OH)_3_ layer maintains a constant thickness of ~5.0 Å [the distance between the outermost edge of the atom columns in the topmost layer and the Al_2_O_3_/Al(111) interface], while the inner Al_2_O_3_ layer thickens by inward migration of the atomically rough Al_2_O_3_/Al(111) interface. This indicates that H_2_O molecules on the pristine Al(111) dissociate dominantly into OH^−^ and H^+^ to result in the formation of the Al(OH)_3_ layer. Thereafter, the observed inward Al_2_O_3_ film growth is induced by the inward diffusion of O atoms derived from the dissociative H_2_O adsorption on the Al(OH)_3_ overlayer. The randomly arriving O atoms at the Al_2_O_3_/Al(111) interface are incorporated into the Al lattice at any site of the interface (*10*, *25*), thereby resulting in the atomically rough Al_2_O_3_/Al(111) interface morphology. The schematic atomic structure of the Al(OH)_3_/Al_2_O_3_ bilayer configuration is illustrated in Fig. 1H.

Figure 1I shows the measurement of the thickness evolution of the Al(OH)_3_/Al_2_O_3_ bilayer film and the inward displacement distance of the Al_2_O_3_/Al(111) interface over the course of time, both of which display an initial fast growth followed by notably slower thickening to a limiting-thickness regime. This self-limiting growth behavior can be fitted well with the logarithmic growth law of the Cabrera-Mott model (Fig. 1I), where the difference between the two self-limiting growth curves differs by ~5.0 Å that corresponds to the thickness of the upper Al(OH)_3_ layer that stays constant with time once formed. As stipulated by the Cabrera-Mott theory, the self-limiting oxide film growth results from electron tunneling from the metal through the oxide film to adsorbed O that leads to a self-generated electric field across the oxide film, which lowers the energy barrier for ion migration across the oxide film and makes the oxide film growth possible at a low temperature (where the thermally driven diffusion is negligible) (*26*). Because the tunneling current decreases exponentially with increasing the thickness of the oxide film, the oxidation stops at a limiting thickness of the oxide film. The magnitude of the self-generated electric field depends on the surface coverage of O (or OH derived from dissociative H_2_O adsorption) to accept the tunneling electrons, where a higher O (or OH) coverage corresponds to a stronger electric field and thus a larger limiting thickness (*27*–*29*). This is consistent with our ex situ TEM observations, showing a larger limiting thickness (~5.7 nm) of the Al(OH)_3_/Al_2_O_3_ bilayer oxide film formed in deionized (DI) water.

**Fig. 2.**
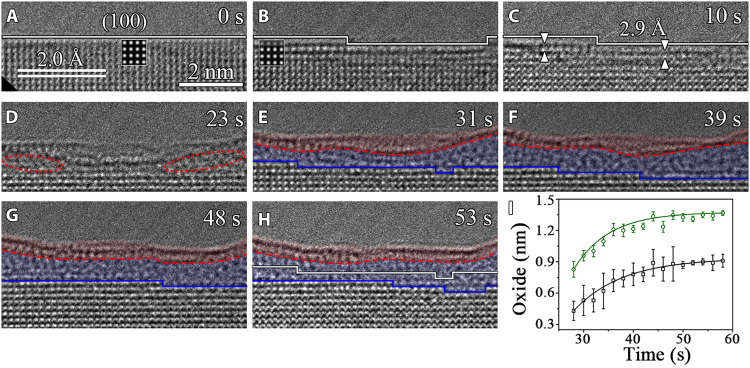
Water vapor–induced surface passivation of pristine Al(100). (**A** to **H**) Time-sequence HRTEM images (movie S2 captured by a direct detection camera) showing the Al(OH)_3_/Al_2_O_3_ bilayer film growth at 298 K in pH_2_O ≈ 8.5 × 10^−5^ torr. The regions marked by solid white lines show weakened image contrast-induced by the extraction of Al atoms from outermost layer upon the surface adsorption of H_2_O molecules. The insets in (A) and (B) are simulated HRTEM images of the perfect Al lattice and the Al lattice with atomic vacancies in the topmost layer. The dashed red and solid blue lines outline the Al(OH)_3_/Al_2_O_3_ and Al_2_O_3_/Al(100) interfaces, respectively. The solid white line in (H) is the superimposed trace of the position and profile of the Al_2_O_3_/Al(100) interface at *t* = 31 s in (E). (**I**) Time evolution of the Al(OH)_3_/Al_2_O_3_ bilayer film thickness (green) and the Al_2_O_3_/Al(100) interface displacement distance (black), where the error bars represent SD uncertainties based on multiple measurements on the in situ TEM images.

Figure 2 presents time-sequenced HRTEM images illustrating the evolution of the Al(100) surface viewed along the [001] zone axis, in the course of the H_2_O exposure at 8.5 × 10^−5^ torr and 298 K. The freshly produced Al(100) surface is atomically flat, and the interplanar spacing (~2.0 Å) matches well the interplanar spacing of Al(200) planes (Fig. 2A). The H_2_O exposure results in the extraction of Al atoms from the topmost layer, as indicated by the weakened lattice contrast in the region marked by the solid white line in Fig. 2B and simulated HRTEM image (Fig. 2B, inset). Upon the continued H_2_O exposure, more Al atoms are extracted from the topmost layer, thereby resulting in the formation of a monolayer-deep depression (pit) and hydroxylation of the two atomic planes at the bottom of the depression, as indicated by the increased interplanar spacing from 2.0 Å of the pristine Al(100) lattice to 2.9 Å in the region marked by the white triangles in Fig. 2C. Meanwhile, Fig. 2C also shows that the surface region adjacent to the depression undergoes the lattice spacing expansion to 2.9 Å, indicating its direct hydroxylation without abstracting Al atoms from that surface region. This surface pitting process transforms the initially atomically flat surface into an uneven morphology of the hydroxylated surface, as shown in Fig. 2 (D to H). Similar to the Al(111) surface, the hydroxylation reaction on the Al(100) surface from the longer H_2_O exposure also results in Al(OH)_3_/Al_2_O_3_ bilayer film growth, and the top Al(OH)_3_ layer maintains its crystalline state and the constant thickness (~5.0 Å), while the inner amorphous Al_2_O_3_ layer grows to a limiting thickness via the inward movement of the Al_2_O_3_/Al(100) interface. Figure 2I shows the thickness evolution of the Al(OH)_3_/Al_2_O_3_ bilayer film and the inward displacement distance of the Al_2_O_3_/Al(100) interface, both of which display a self-limiting growth behavior and can be fitted well with the logarithmic growth law of the Cabrera-Mott model. In contrast to the Al_2_O_3_/Al(111) interface that remains atomically rough during the Al_2_O_3_ growth (Fig. 1), the Al_2_O_3_/Al(100) interface consists of flat (100) terraces and monoatomic ledges. Our in situ TEM observation shows that the interfacial Al_2_O_3_ growth occurs via lateral flow of ledges along the interface, and these ledges are supplied by repeated nucleation at the Al_2_O_3_/Al(100) interface through interface O embedment into the Al lattice (Fig. 2 (E to H).

**Fig. 3.**
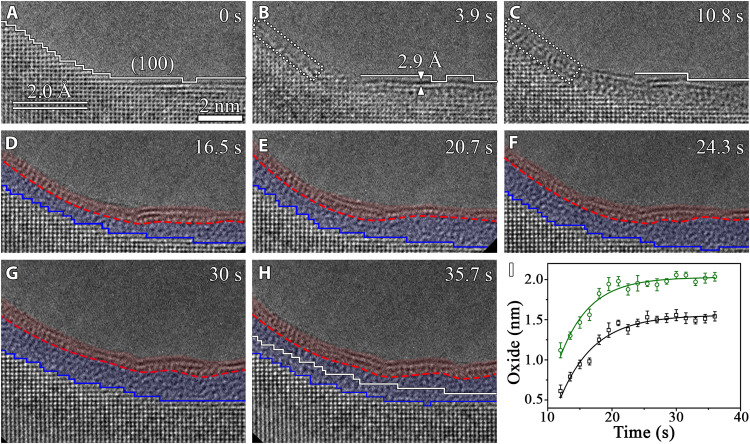
Water vapor–induced surface passivation of a corner region consisting of a flat (100) facet and a stepped facet. (**A** to **H**) Time-sequence HRTEM images (movie S3) showing the Al(OH)_3_/Al_2_O_3_ bilayer film growth at 298 K in pH_2_O ≈ 3.5 × 10^−4^ torr. The dashed white rectangles mark the H_2_O adsorption–induced direct disordering of the stepped facet before its Al(OH)_3_ outer layer is established. The dashed red and solid blue lines mark the Al(OH)_3_/Al_2_O_3_ and Al_2_O_3_/Al interfaces, respectively. The solid white line in (H) is the superimposed trace of the position and profile of the Al_2_O_3_/Al interface at *t* = 16.5 s in (D). Scale bar, 2 nm (A to H). (**I**) Time evolution of the Al(OH)_3_/Al_2_O_3_ bilayer film thickness (green) and the Al_2_O_3_/Al interface displacement distance (black), where the error bars represent SD uncertainties based on multiple measurements on the in situ TEM images.

Figure 3 presents real-time HRTEM images displaying the hydroxylation reaction of an intersection region between flat (100) and a highly stepped facet exposed to 3.5 × 10^−4^ torr of H_2_O vapor at 298 K. The H_2_O adsorption results in the abstraction of Al atoms from the outermost layer of the (100) facet, as indicated by the weakened image contrast marked by the solid white lines in Fig. 3 (B and C). This is accompanied by the surface hydroxylation of the topmost two atomic layers, as indicated by the lattice expansion from 2.0 Å of the pristine Al(100) lattice to 2.9 Å. By contrast, the adjacent stepped facet does not show obvious Al abstraction from the topmost surface layer. Instead, the H_2_O adsorption leads to the disordering of the two topmost two atomic layers (the region marked by the dashed white rectangle in Fig. 3B), followed by their transition to the crystalline Al(OH)_3_, as indicated by the restored lattice contrast in the region marked by the dashed white rectangle in Fig. 3C (see more example in fig. S2). Upon continued H_2_O exposure, the formation of amorphous Al_2_O_3_ occurs underneath the Al(OH)_3_ overlayer that remains a constant thickness and stays adherent to the Al_2_O_3_ layer despite the uneven surface morphology (Fig. 3, D to H). The Al_2_O_3_ layer thickens via the oxide growth at the Al_2_O_3_/Al interface, where the (100) interface growth occurs via the nucleation and lateral flow of interfacial ledges, whereas the adjacent stepped interface maintains atomically rough during the Al_2_O_3_ growth, as indicated by the blue lines in Fig. 3 (D to H). Similar to the (111) and (100) surfaces, both the thickness evolution of the Al(OH)_3_/Al_2_O_3_ bilayer and the Al_2_O_3_/Al interface displacement distance can be fitted well with the logarithmic growth law of the Cabrera-Mott model, as shown in Fig. 3I.

### H_2_O vapor–induced Al(OH)_3_ formation on amorphous Al_2_O_3_

**Fig. 4.**
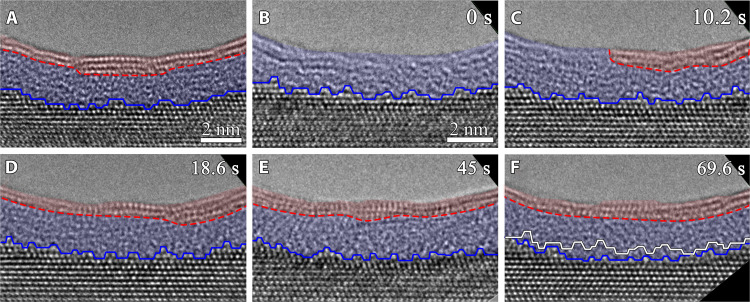
Water vapor–induced Al(OH)_3_ formation on an amorphous Al_2_O_3_ overlayer. (**A**) HRTEM image of a Al(OH)_3_/Al_2_O_3_ bilayer film on Al(111). (**B**) The Al(OH)_3_ outer layer is largely sputtered off using the condensed electron beam. (**C** to **F**) Time-sequence HRTEM images showing the recovery of the Al(OH)_3_/Al_2_O_3_ bilayer film configuration upon the hydroxylation of the amorphous Al_2_O_3_ at 298 K in pH_2_O ≈ 3.5 × 10^−4^ torr. The dashed red and solid blue lines mark the Al(OH)_3_/Al_2_O_3_ and Al_2_O_3_/Al(111) interfaces, respectively. The solid white line in (F) is the superimposed trace of the position and profile of the Al_2_O_3_/Al interface at *t* = 0 s in (B).

The in situ TEM observations shown above demonstrate that the H_2_O exposure results in the Al(OH)_3_/Al_2_O_3_ bilayer oxide growth on the Al surfaces. The Al(OH)_3_ overlayer maintains a crystalline state at a constant thickness, while the underlying amorphous Al_2_O_3_ layer grows inward at the Al_2_O_3_/Al interface. Figure 4 shows in situ TEM observations that further confirm this inherent feature of forming a crystalline Al(OH)_3_ overlayer from the H_2_O-induced surface hydroxylation at 298 K. As shown in Fig. 4A, the Al(OH)_3_/Al_2_O_3_ bilayer is visible from the H_2_O exposure onto a clean Al(111) surface, where the Al(OH)_3_/Al_2_O_3_ and Al_2_O_3_/Al interfaces are indicated by the dashed red and solid blue lines, respectively. The crystalline Al(OH)_3_ overlayer can be purposely sputtered off inside the TEM with a condensed electron beam > ~60,000 *e* Å^−2^ s^−1^ (in the presence of H_2_O vapor), leaving behind largely the amorphous Al_2_O_3_ layer with a thinner thickness, as illustrated in Fig. 4B. Soon after the electron beam is spread, the crystalline feature starts to rebuild locally on the amorphous Al_2_O_3_ layer, such as in the region marked by the dashed red line in Fig. 4C. This trend becomes more evident, and the outermost amorphous region of the entire surface gradually transforms into the crystalline state of Al(OH)_3_. This resumes the Al(OH)_3_/Al_2_O_3_ bilayer configuration, in which the inner Al_2_O_3_ layer still maintains the amorphous state. The recovered Al(OH)_3_ layer also demonstrates the dissociation of H_2_O molecules on the amorphous Al_2_O_3_ into OH and H to result in the formation of the Al(OH)_3_ outer layer. This is indicated by the increased overall thickness of the Al(OH)_3_/Al_2_O_3_ bilayer film via the inward Al_2_O_3_ growth along the atomically rough Al_2_O_3_/Al(111) interface, as shown by tracing the movement of the Al_2_O_3_/Al(111) interface depicted in Fig. 4F.

The above in situ TEM results of the Al(OH)_3_/Al_2_O_3_ bilayer film growth are confirmed from various experiments conducted on different regions of multiple samples (figs. S2 to S5). When working with in situ TEM observations (Figs. 1 to 4 and fig. S2), the electron beam effects such as radiolysis and local heating need to be carefully examined. It was reported that a strong electron flux > ~12,000 *e* Å^−2^ s^−1^ can induce homogeneous crystallization inside the amorphous Al_2_O_3_ film (*9*), which differs from the Al(OH)_3_/Al_2_O_3_ bilayer configuration formed from the H_2_O exposure shown here at the electron flux of ~8300 *e* Å^−2^ s^−1^. We also perform both the “low-dose” and “blank-beam” experiments to ensure that an intrinsic behavior is studied. With the use of the electron counting mode of a direct detection camera at the electron flux of <2300 *e* Å^−2^ s^−1^ to minimize the electron irradiation effects while maintaining the lattice resolution, the in situ TEM imaging clearly shows the Al(OH)_3_/Al_2_O_3_ bilayer film growth (Fig. 2 and fig. S4). In addition, blank-beam experiments are also performed and demonstrate the Al(OH)_3_/Al_2_O_3_ bilayer film formation on Al surfaces from the H_2_O exposure in the dark (fig. S5). Ex situ TEM observations are also performed to confirm the formation of the Al(OH)_3_/Al_2_O_3_ bilayer structure outside the ETEM conditions. This is illustrated by ex situ TEM observations of an Al foil that is directly immersed into DI liquid water, showing the formation of a bilayer structure consisting of a crystalline-like top layer and an inner amorphous layer (fig. S6), consistent with the Al(OH)_3_/Al_2_O_3_ bilayer structure observed from the ETEM experiments. In addition, the effect of possible background gas molecules (such as H_2_, O_2_, water vapor, and carbonyl) in the TEM column is found to be negligible. This is confirmed by in situ TEM imaging, showing that the Al surfaces under vacuum (base pressure of 8 × 10^−8^ torr) maintain oxide-free for a relatively long period of time inside the TEM before flowing H_2_O vapor into the sample region (fig. S7). The effect of any residual O_2_ in the TEM column is also confirmed to be insignificant, as shown by comparative in situ HRTEM imaging of the Al surfaces in 3.5 × 10^−5^ torr of H_2_O and O_2_, respectively, where the former leads to the Al(OH)_3_/Al_2_O_3_ bilayer oxide film (Figs. 1 to 4), whereas the latter only forms an amorphous Al_2_O_3_ layer (fig. S8). All the in situ and ex situ TEM observations confirm that the Al(OH)_3_/Al_2_O_3_ bilayer film formation is an intrinsic feature for the H_2_O-induced surface passivation, irrespective of the electron beam irradiation, crystallographic orientations, and surface defects.

It can be also noted from the above in situ TEM imaging that the crystalline-like Al(OH)_3_ can readily form on the amorphous Al_2_O_3_ layer, irrespective of the surface morphology and orientation of the Al substrate. This easy formation of the crystalline Al(OH)_3_ phase can be attributed to the underlying amorphous Al_2_O_3_ layer, for which there is the absence of the epitaxial strain in the Al(OH)_3_ overlayer. As a result, the crystalline phase of Al(OH)_3_ is thermodynamically more favored than its amorphous counterpart. This is in contrast to the Al_2_O_3_/Al interface, where the crystalline Al substrate makes the formation of the crystalline Al_2_O_3_ unfavorable due to their large lattice misfit. Instead, the amorphous Al_2_O_3_ becomes more favorable to minimize the Al_2_O_3_/Al interface strain. Such an interface effect is in good accordance with the thermodynamics calculations, showing that the formation of the ultrathin amorphous Al_2_O_3_ film is more favored than its crystalline counterpart from the oxidation of Al surfaces (*30*, *31*).

### AP-XPS and RGA measurements of the Al surface passivation in H_2_O vapor

**Fig. 5.**
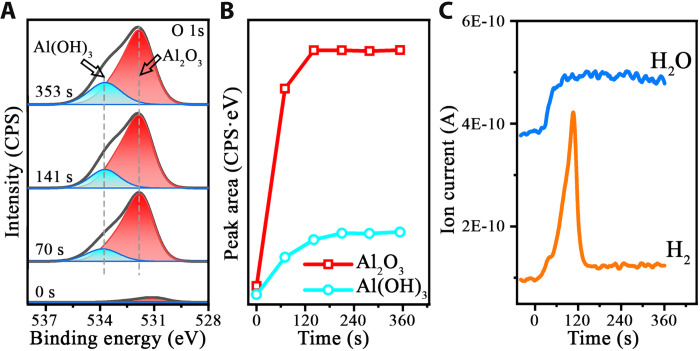
AP-XPS and RGA measurements of the passive oxide film formation on Al(111). (**A**) Time-resolved photoemission spectra and intensity counts per second (CPS) of the O 1s core-level region obtained during the exposure of pristine Al(111) at 298 K to 1 × 10^−5^ torr of water vapor, where the faint peak intensity at 0 s can be attributed to the residual oxygen that cannot be completely removed by sputtering and annealing. (**B**) Time evolution of the integrated intensity of the Al_2_O_3_ and Al(OH)_3_ components. (**C**) Coordinated RGA measurements of the evolution of the feeding H_2_O (blue) and H_2_ production (orange).

To cross-validate the in situ TEM observations, complementary ambient-pressure XPS (AP-XPS) is used to chemically confirm the formation of the Al(OH)_3_/Al_2_O_3_ bilayer film from the H_2_O vapor exposure. Figure 5A illustrates time-resolved O 1s XPS spectra while exposing a clean Al(111) surface at 298 K to 1 × 10^−5^ torr of H_2_O vapor, showing the appreciable O 1s peak intensity after the H_2_O exposure. The O 1s exhibits the asymmetry to the high binding energy side and can be deconvoluted into two peaks with the binding energies of 532.1 and 533.8 eV. The peak at 532.1 eV is attributed to the O in Al─O bonds of Al_2_O_3_, whereas the other peak at 533.8 eV is consistent with the O in Al─OH bonds of Al(OH)_3_ (*29*, *32*–*34*). This corroborates well with the Al 2p spectra (fig. S9) that can be deconvoluted into metallic (Al^0^) at 72.8 eV and oxidized Al^3+^ at 75.5 eV, respectively. It is worth mentioning that the binding energies for Al in Al_2_O_3_ and Al(OH)_3_ are 75.6 and 75.2 eV, respectively, which are too close to differentiate from the XPS measurements (*29*, *34*, *35*). Figure 5B corresponds to the integrated intensity evolution of O 1s spectra as a function of time, showing the dominant peak intensity by the Al_2_O_3_ component and the rapid peak intensity growth to the saturated level for both the Al_2_O_3_ and Al(OH)_3_ components. This is consistent with the in situ TEM imaging of the self-limiting Al oxide film growth that is dominated by the Al_2_O_3_ growth at the Al_2_O_3_/Al interface, while the upper Al(OH)_3_ layer maintains a constant thickness. As revealed from the in situ TEM imaging (Figs. 1 to 3), the Al(OH)_3_ formation takes place within the first ~20 s of the H_2_O exposure before the Al_2_O_3_ growth. This sequential formation of Al(OH)_3_ and Al_2_O_3_ cannot be detected readily by AP-XPS because of its long data acquisition time (~1 min) that results in the temporal summation of the overall signals from the probed surface area (~300 μm).

Figure 5C illustrates the coordinated residual gas analyzer (RGA) measurements of the gas composition evolution during flowing the H_2_O vapor in the AP-XPS chamber. The measurement shows a spike of the H_2_ production, while the feeding H_2_O vapor maintains at the constant pressure. The RGA results show that the H_2_ product amount gradually increases to the maximum and then drops to zero after ~120 s of the H_2_O exposure, which is correlated well with the time period of reaching the limiting thickness of the oxide film measured by the O 1s spectra (Fig. 5A). Because the maximum H_2_ production takes place after ~100 s of the H_2_O dosing (Fig. 5C), this suggests that the Al(OH)_3_ surface termination is more reactive toward the H_2_ formation than the pristine metallic Al surface. The termination of the H_2_ production is attributed to the reached limiting thickness of the oxide film, for which the Al(OH)_3_ surface restores its stoichiometry and thus loses its reactivity toward dissociative H_2_O adsorption, as described in our density functional theory (DFT) modeling below. It is also worth mentioning that the penetration of atomic H produced from the dissociative H_2_O adsorption into the deeper region below the Al(OH)_3_ layer can be negligible. This can be evidenced by the AP-XPS measurements (Fig. 5, A and B), which chemically confirm the dominant presence of the O peak compared to the OH peak. In addition, the coordinated RGA measurements show the production of gaseous H_2_ before reaching the limiting thickness of the passivating film (Fig. 5C), indicating that the H from the dissociative H_2_O adsorption combines into molecular H_2_ that desorbs from the surface rather than penetrates to the Al_2_O_3_ region for hydrolysis to occur.

### Atomistic modeling of the passive oxide film formation

**Fig. 6.**
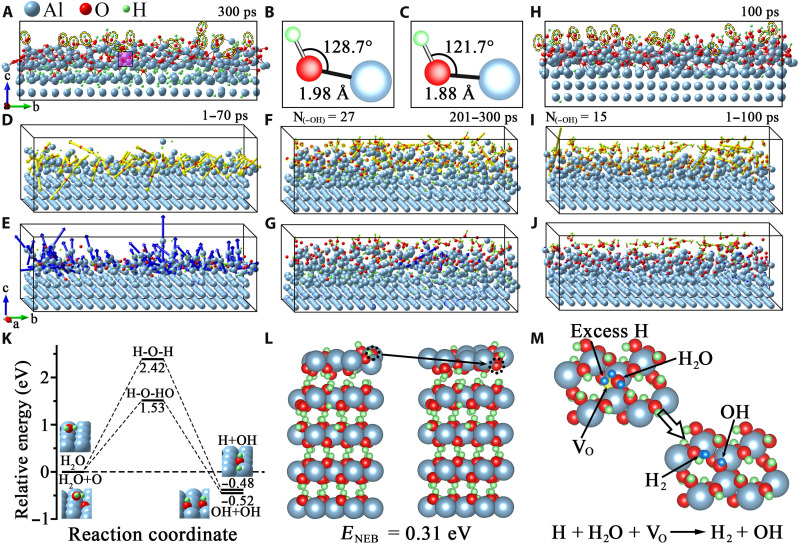
ReaxFF MD and DFT simulations of water vapor–induced surface passivation. (**A**) Snapshot of MD simulations of a Al(100) surface after 300 ps of H_2_O exposure at 298 K (movies S5 to S7). (**B**) Al─O─H bonding configuration of the purple-shaded region in (A) and its comparison to that of bulk Al(OH)_3_ in (**C**). (**D** and **E**) Displacement vectors of O and Al atoms between 1 and 70 ps of H_2_O exposure. (**F** and **G**) Displacement vectors of O and Al atoms between 201 and 300 ps of H_2_O exposure. (**H**) Snapshot of MD simulation of a preoxidized Al(100) surface after 100 ps of H_2_O exposure at 298 K (movie S8). (**I** and **J**) MD simulations of H_2_O-induced hydroxylation and displacement vectors of O and Al atoms between 1 and 100 ps of H_2_O exposure of an Al_2_O_3_ overlayer formed by the exposure of Al(100) at 298 K to O_2_. The dashed yellow circles in (A) and (H) mark the formed hydroxyl on the surface. (**K** and **L**) Nudged elastic band (NEB) modeling of the dissociation pathways and energy barriers for H_2_O dissociation on Al(111) and the migration of surface O to an O vacancy site in the subsurface of the bulk-truncated Al(OH)_3_(001), respectively. (**M**) DFT modeling of the dissociation pathway for the adsorbed H_2_O on the defective Al(OH)_3_(001) surface with the presence of an O vacancy and excess H at the surface.

The in situ TEM observations and XPS results above are mutually consistent in providing strong evidence of the H_2_O-induced Al(OH)_3_/Al_2_O_3_ bilayer film growth. The observed inward Al_2_O_3_ growth at the Al_2_O_3_/Al interface along with the constant thickness of the top Al(OH)_3_ layer suggests that the oxidation is dominated by the inward migration of O from the top Al(OH)_3_ layer to the Al_2_O_3_/Al interface and the outward Al diffusion is negligible. To shed light on the microscopic mechanism of the bilayer film growth, reactive force-field (ReaxFF) molecular dynamics (MD) simulations are performed to examine the surface reaction between Al surfaces and H_2_O vapor. Figure 6 (A to G) shows the simulation snapshots captured at different H_2_O exposure times of the Al(100) surface. As shown in the snapshot of 300 ps of H_2_O exposure (Fig. 6A), Al─OH bonds are formed in the oxide layer, which indicates the dissociation of H_2_O into OH^−^ and H^+^ to result in the surface hydroxylation, consistent with the in situ TEM observations of the direct Al(OH)_3_ formation on pristine Al surfaces (Figs. 1 and 2). Figure 6A also shows the penetration of atomic H into the Al lattice, which is caused by the use of the Berendsen thermostat (*36*) to accelerate the notably slow dissociation of H_2_O molecules at room temperature in the MD simulations (*37*–*39*). This approach was used in previous studies to investigate the interactions between H_2_O molecules and the Al surface (*36*, *40*). Figure 6B illustrates a presentative Al─(OH) bond configuration resulting from the H_2_O exposure, showing that the Al─OH bond lengths range from 1.90 to 1.98 Å, and the Al─O─H bond angles fall into the range of 128.4° to 176.9°, which are close to the corresponding bond length (1.88 to 1.97 Å) and bond angle (105.1° to 121.7°) in bulk Al(OH)_3_ (Fig. 6C). However, it also needs to be mentioned that the produced Al─OH bond angles fall into a larger range than that of bulk Al(OH)_3_ because the Al─OH bonds in the surface oxide layer are more defective than the ordered arrangement of bulk Al(OH)_3_.

Figure 6 (D and E) shows the displacement vectors of O and Al atoms between 1 and 70 ps, which correspond to the initial-stage surface hydroxylation by H_2_O molecules. As can be seen in Fig. 6D, nearly all the displacement vectors of O atoms point inward, which indicates inward migration of O atoms toward the Al substrate. In contrast, the displacement vectors of Al atoms in the oxygenated region largely point outward (Fig. 6E), indicating the outward relaxation of Al atoms due to the embedment of O atoms into the Al lattice at the Al_2_O_3_/Al interface. Figure 6 (F and G) shows the displacement vectors of O and Al atoms between 201 and 300 ps, corresponding to a relatively later stage of the H_2_O exposure. As shown in Fig. 6F, the O atoms still display the inward displacements, which indicates the embedment of more O atoms into the Al lattice and corresponds to the tendency of forming an inner Al_2_O_3_ layer. The number of Al─OH bonds remains nearly constant after 220 ps of H_2_O exposure. This indicates the trend of approaching a constant thickness of the Al hydroxide outer layer, agreeing well with the in situ TEM observations of the constant thickness of the Al(OH)_3_ layer (Figs. 1 to 3 and fig. S2 and S4). The displacement vectors of Al atoms (Fig. 6G) show slight outward relaxations induced by the continued O embedment into the Al substrate.

To further elucidate the surface nature of the Al hydroxide formation in H_2_O, ReaxFF MD simulations of the surface oxidation of Al(100) are performed sequentially, first in O_2_ and then switching to H_2_O. That is, pristine Al(100) is first exposed to O_2_ to result in the formation of an amorphous Al oxide layer after 100 ps of the canonical relaxation under the conditions of a constant particle number (N), constant volume (V), and constant temperature (T) (NVT). The O_2_-generated Al(100) slab is then used to simulate the dissociative adsorption of H_2_O molecules via the same procedure as that in the H_2_O exposure simulation mentioned above. Figure 6H displays a simulation snapshot after 100 ps of the H_2_O exposure, showing the formation of Al─OH bonds dominated in the outermost surface region. This is consistent with the in situ TEM observation (Fig. 4), showing the formation of a Al(OH)_3_ layer on the amorphous Al_2_O_3_ layer from the subsequent H_2_O exposure. Figure 6 (I and J) illustrates the displacement vectors of O and Al atoms between 0 and 100 ps, corresponding to the H_2_O exposure to the O_2_-generated Al(100) slab. As shown in Fig. 6I, the O atoms still show the inward displacements, which indicates the continued incorporation of dissociated O atoms into the Al lattice from the surface adsorbed H_2_O, thereby resulting in the inward migration of the Al_2_O_3_/Al interface. Figure 6J displays the displacement vectors of Al atoms showing the slight outward relaxations due to the continued O embedment into the Al substrate.

Last, we illustrate the atomic origin leading to the Al(OH)_3_/Al_2_O_3_ bilayer film growth in H_2_O. Our in situ TEM observations and MD simulation above have shown that the H_2_O-induced surface passivation occurs via a two-stage process: first the Al(OH)_3_ formation via H_2_O dissociation into OH groups for the Al(OH)_3_ formation followed by Al_2_O_3_ growth through inward O diffusion. This implies a difference in the reaction pathway for the pristine Al surface and the Al(OH)_3_ terminated surface. DFT calculations are performed to evaluate this difference in the dissociation pathways of H_2_O molecules. As shown in Fig. 6K, our DFT calculations show that it is energetically favorable for the dissociation of a H_2_O molecule on Al(111) into OH^−^ and H^+^ by overcoming an energy barrier of ~2.42 eV, and the presence of preadsorbed O on the surface (formed due to the presence of any residual O_2_ in the surrounding) can reduce the energy barrier to ~1.53 eV. This is in good accordance with other DFT studies showing the preferential OH formation of H_2_O molecules on pristine Al surfaces (*41*–*43*).

For the Al(OH)_3_ surface, our DFT calculations show that the perfect, stoichiometric surface does not show any activity toward dissociative H_2_O adsorption. As informed from our in situ TEM observations, the Al_2_O_3_ growth occurs at the Al_2_O_3_/Al interface, which requires the supply of O ions from the top Al(OH)_3_ layer to the inner interface. The diffusion energy barrier for such inward migration of O ions from the Al(OH)_3_ surface to an O vacancy site in the subsurface is calculated to be 0.31 eV (Fig. 6L). This small diffusion energy barrier suggests that the inward diffusion of O ions across the Al(OH)_3_ layer is kinetically achievable. Therefore, this inward Al_2_O_3_ growth results in O vacancies (V_O_) and excess H at the Al(OH)_3_ surface, which may facilitate the dissociative adsorption of H_2_O. Our DFT calculations show that the molecular H_2_O placed slightly above the O vacancy site results in its spontaneous dissociation into H^+^ and OH^−^, where the former bonds with adjacent excess H to form a H_2_ molecule that desorbs from the surface, whereas the latter occupies the original V_O_ site (Fig. 6M). The DFT modeling also explains why the Al(OH)_3_ layer stays as the top layer because it is in direct contact with the H_2_O vapor, where the dissociative H_2_O adsorption provides OH species to maintain the Al(OH)_3_ structure in addition to the H_2_ production. The process of the inward O migration and H_2_O dissociative adsorption at the V_O_ sites repeats itself until the oxide film reaches its limiting thickness, at which the termination of the Al_2_O_3_ growth leads to the restoration of the surface stoichiometry of the Al(OH)_3_ layer. As a result, the Al(OH)_3_ surface loses its reactivity toward further dissociative H_2_O adsorption.

## DISCUSSION

The wide use of Al is attributed to its ability to form a passivation layer that is typically assumed to have an amorphous structure in nature (*44*–*47*). As shown above, our in situ TEM imaging clearly demonstrates that the passivating layer on Al surfaces in water consists of a crystalline Al(OH)_3_ outer layer and an amorphous Al_2_O_3_ inner layer. This Al(OH)_3_/Al_2_O_3_ bilayer configuration not only differs microstructurally from the surface passivation in dry O_2_ that results in a single layer of amorphous Al_2_O_3_ (see the example in fig. S8) but also alters the passivating film growth mechanism from both inward O diffusion and outward Al diffusion in O_2_ (*10*) to the inward O diffusion in H_2_O. The inward O diffusion for Al_2_O_3_ growth at the Al_2_O_3_/Al interface results in V_O_ and excess H at the crystalline Al(OH)_3_ overlayer, which promotes the H_2_ production from the dissociative H_2_O adsorption. This insight may have important implications for the design of materials in clean-energy and environmental applications. For instance, Al has been proposed for onboard vehicular H_2_ storage for making clean H_2_ from the reaction between Al and H_2_O (*48*–*50*). Our results shown above provide the mechanistic insight into this reaction by identifying the important role of the Al(OH)_3_ formation for the H_2_ production from H_2_O. That is, the Al(OH)_3_ overlayer serves as a catalyst to promote the reaction pathway of H + H_2_O + V_O_ → OH + H_2_ at ambient temperature. On the other hand, the corrosion of metallic materials in humid environments is encountered in daily life due to the ubiquity of water. The atomistic mechanisms identified from the passivating film formation on Al surfaces in H_2_O may find applicability to understand the microscopic mechanisms controlling the surface passivating dynamics of other metals under humid conditions.

Using a combination of in situ TEM and atomistic modeling, we provide direct evidence that the surface passivation of Al surfaces in water vapor results in the formation of an Al(OH)_3_/Al_2_O_3_ bilayer film. The Al(OH)_3_ outer layer has a crystalline structure and maintains a constant thickness, while the inner amorphous Al_2_O_3_ layer grows at the Al_2_O_3_/Al interface to a limiting thickness. Such bilayer passivating film growth is related to the two-stage passivation reaction: first, the H_2_O dissociation into OH + H on the pristine Al surface to form the Al(OH)_3_ overlayer and, then, into OH + H_2_ on the O-deficient Al(OH)_3_ overlayer due to inward O diffusion to the Al_2_O_3_/Al interface. These fundamental insights demonstrate the tunability of the dissociation pathways of H_2_O molecules and have practical implications not only in controlling the microscopic processes of the passivating film growth but also in clean H_2_ production from H_2_O catalyzed by the spontaneously formed Al hydroxide overlayer on Al.

## MATERIALS AND METHODS

### Sample preparation

Thin Al foils with a nominal thickness of ~50 nm are prepared using the focused ion beam (FIB) lift-out technique (FEI Helios Nanolab 600) and the NanoMill system. To minimize potential surface damage and Ga contamination, the ion beam with a low voltage (5 kV) and current (9 pA) is used to do the final trimming of the surface of the sliced sample during the FIB process. Thereafter, the Al slice is loaded on a Mo Omniprobe Lift-out grid and further polished by the NanoMill TEM specimen preparation system with a lower voltage (900 V) and current (80 pA) of Ar^+^ ions to remove the possible surface damage and contamination. The as-prepared Al thin foils are then examined by HRTEM imaging and chemical analyses, confirming that the structure damage and contamination from the FIB process are negligible (figs. S10 and S11).

### In situ HRTEM experiments

In situ TEM experiments are performed using an image-corrected environmental TEM (FEI Titan 80-300) operated at 300 kV, which is equipped with a differential pumping system. The microscope has a spatial resolution of 0.8 Å in the HRTEM mode. Atomically clean Al surfaces are obtained using a condensed electron beam inside the TEM column to sputter off native oxide and generate well-defined facets with a thickness (~50 nm) (*9*). These freshly generated facets are oxide-free and ideal for in situ TEM observations of water vapor–induced surface passivation from the beginning (see more details in fig. S1). The in situ HRTEM images are captured with a positive Cs value (1 to 3 μm). Complete removal of the native oxide and surface cleanliness are confirmed by HRTEM imaging, electron diffraction, and electron energy loss spectroscopy. Water vapor is then introduced into the sample area through a leak valve to oxidize the Al foils at a given temperature and gas pressure. In situ TEM observations of the passivation process are made in the cross-sectional views by imaging along surface facets. The in situ TEM movies are drift-corrected to ensure the same sample area in the field of view.

### HRTEM image simulations

The DFT-relaxed atomic structure models of Al and Al(OH)_3_ are used as input files for HRTEM image simulations. HRTEM image simulations are performed using the multislice method with the parameters carefully matched to the experimental conditions (accelerating voltage, 300 keV; the spherical aberration, 0.001 mm; defocus, −8 nm; and thickness, 28 nm). The frozen phonon model is applied to reduce the elastic scattering and increase the background intensity.

### In situ AP-XPS experiments

XPS measurements are performed within an ultrahigh vacuum system. The system is equipped with an XPS spectrometer (SPECS Phoibos 150 MCD analyzer) with a delay-line detector, and an Ar-ion sputtering gun. The chamber has a base pressure of 1.5 × 10^−10^ torr. Al-Kα x-ray radiation is used for the XPS measurements. The Al(111) single crystal is a “top-hat” disk (1-mm thick and 8 mm in diameter), purchased from Princeton Scientific Corp., cut to within 0.1° to the (111) crystallographic orientation, and polished to a mirror finish. The crystal is cleaned by cycles of Ar^+^ bombardment at 298 K and annealing to 700 K. Water vapor (purity, 99.9999%) is introduced to the system through a leak valve, and the sample is oxidized at 298 K with a water vapor pressure of 1 × 10^−5^ torr.

### ReaxFF MD simulations

MD simulations are performed using the LAMMPS code (*45*). The bonding, angle, and torsion between the Al, H, and O atoms are described using the ReaxFF interatomic potential, which was developed by Van Duin and colleagues (*51*–*53*). A periodic supercell with the Al(001) surface and H_2_O molecules is used to simulate the surface passivation of Al under water vapor. The Al(001) surface is modeled as a 4.04 nm–by–4.04 nm–by–2.83 nm slab, with a vacuum space of 10 nm added above the slab surface to separate each periodic image. First, the Al slab is relaxed with an NPT [a fixed number of particles (N), and constant pressure (P), and temperature (T)] ensemble at 298 K and 1 atm until it reaches a stable structure. Then, 72 H_2_O molecules (16.5 mg/cm^3^) are randomly distributed near the surface, avoiding overlap. Afterward, an NVT ensemble simulation is performed at room temperature on the Al slab and H_2_O molecules for 70 ps with a time step of 0.1 fs, resulting in a stable structure with H_2_O molecules situated on the Al(001) surface. Subsequently, microcanonical ensemble simulations are performed for 100 ps to simulate the water dissociation and surface passivation reactions. Given that the dissociation of H_2_O molecules on the Al slab surface is notably slow, we use the Berendsen velocity scaling thermostat with a damping constant of 100 fs to enhance the reaction rate in the microcanonical ensemble simulation (*39*). We separate the system into two temperature zones: the Al slab, which is treated as a heat sink with a target temperature of 0 K, and the remaining particles, which are accelerated to a target temperature of 1650 K (*36*). We repeat the process of adding H_2_O molecules and conducting subsequent canonical ensemble and microcanonical ensemble simulations three times, a process that we refer to as a three-stage procedure. We use the last trajectory from each stage as the initial structure for the subsequent stage. This approach of accelerating the H_2_O dissociation process was used in the previous study in investigating the interactions between H_2_O molecules and Al nanoparticles (*36*, *39*).

### DFT calculations

DFT calculations are performed using the Vienna Ab initio Simulation Package (*54*, *55*) with the Perdew-Burke-Ernzerhof generalized gradient approximation and projector augmented wave potentials (*56*, *57*). The cutoff energies of 400 and 600 eV are used to calculate the adsorption energies. Their resulting differences are less than 0.1 eV, confirming that the cutoff energy of 400 eV is sufficient for the electronic energy convergence. The (4 × 4 × 1) *K*-point meshes based on Monkhorst-Pack grids are applied for the Brillouin zone integration. The convergence test for the *K*-points mesh is performed by comparing the adsorption energy difference between the (4 × 4 × 1) and (8 × 8 × 1) meshes, which suggests that the (8 × 8 × 1) mesh gives an adsorption energy difference of less than 0.1 eV, indicating that convergence criterion is reached using the (4 × 4 × 1) mesh. Al(111) and Al(OH)_3_ surfaces are constructed by cleaving supercells made from bulk structure. Successive slabs with five atomic layers are separated by a vacuum region of 12 Å. The positions of the atoms in the two bottom layers are fixed, while the positions of the atoms in the top three layers are allowed to relax with the energy convergence less than ~10^−5^ eV and all force components on each of them are less than 0.015 eV/Å. We investigate the adsorption energies of H_2_O, OH, and H species on surfaces and structure evolution for each calculation. Furthermore, the diffusion energy barrier for the incorporation of O on Al(OH)_3_ surface into the subsurface region is calculated using the nudged elastic band method. The atomic structures are visualized using the Visualization for Electronic and Structure Analysis.
