# Supplementary material for: Atomistic mechanisms of water vapor–induced surface passivation
Source: Sci Adv. 2023 Nov 1;9(44):eadh5565. doi: 10.1126/sciadv.adh5565 (PMC10619940; doi:10.1126/sciadv.adh5565)
Supplement: Supplementary file 1 — Supplementary Text Figs. S1 to S11 Legends for movies S1 to S8 [file sciadv.adh5565_sm.pdf]

Science Advances
AAAS

Supplementary Materials for

Atomistic mechanisms of water vapor induced surface passivation

Xiaobo Chen *et al.*

Corresponding author: Guangwen Zhou, gzhou@binghamton.edu

Sci. Adv. **9**, eadh5565 (2023)
DOI: 10.1126/sciadv.adh5565

The PDF file includes:

Supplementary Text
Figs. S1 to S11
Legends for movies S1 to S8

Other Supplementary Material for this manuscript includes the following:

Movies S1 to S8

SI-1. In-situ creation of clean facets by high flux e-beam irradiation

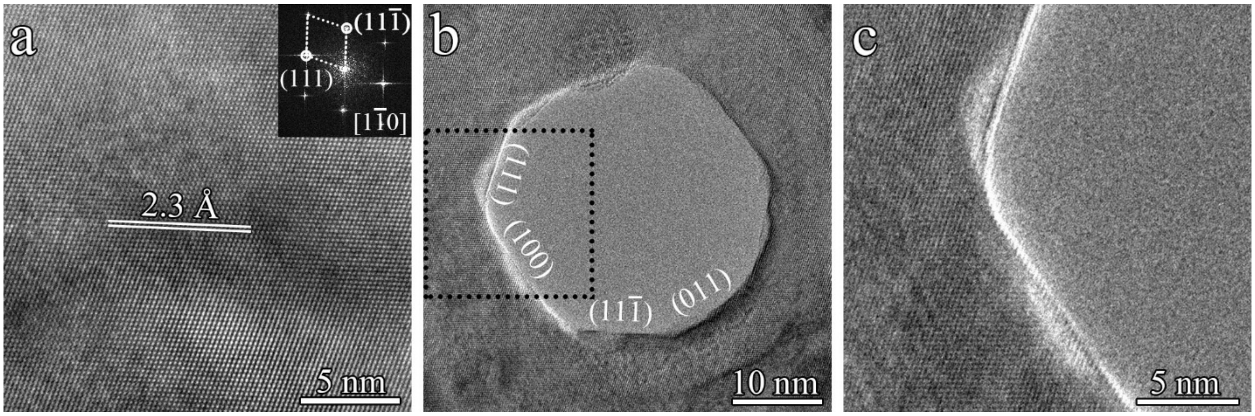

Fig. S1. Creation of faceted holes in an Al foil by high flux e-beam sputtering. (a) HRTEM image of the as-prepared Al foil, viewed along the $[1\bar{1}0]$ zone axis. The inset is the corresponding diffractogram. (b) HRTEM image of the faceted nano-hole drilled by the focused, high flux electron beam. (c) Enlarged view of the area marked by the dashed black rectangle in (b), showing the freshly-generated surface facets.

SI-2. *In situ* observations of water-vapor-induced surface passivation

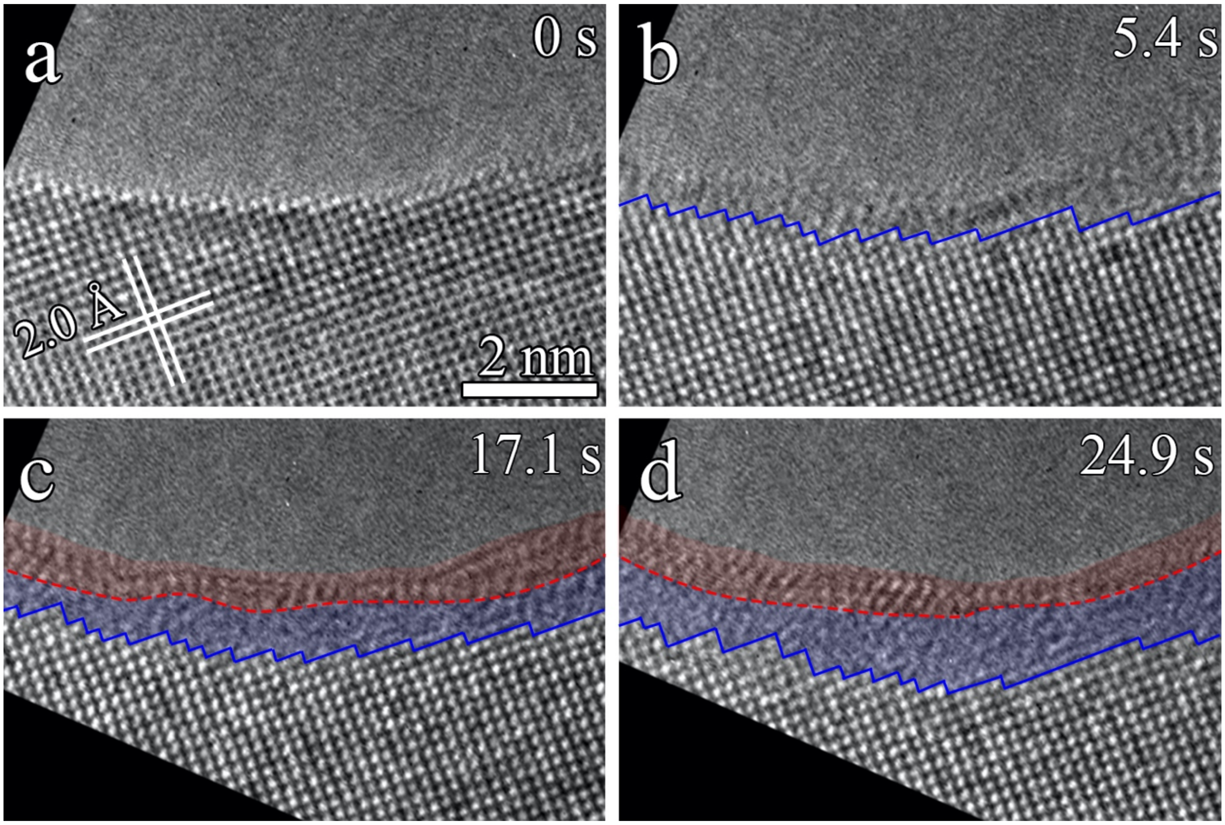

Fig. S2. Water-vapor-induced passive oxide thin film formation on a rough Al surface. (a-d) Time-sequence HRTEM images showing the $Al(OH)_3/Al_2O_3$ bi-layer film growth. Upon the continued H_2O exposure at 298 K in $pH_2O \approx 3.5\times10^{-4}$ Torr, the surface hydroxylation first results in the formation of a crystalline-like $Al(OH)_3$ overlayer, followed by the inward growth of an amorphous Al_2O_3 layer. The dashed red and solid blue lines mark the $Al(OH)_3/Al_2O_3$ and Al_2O_3/Al interfaces, respectively. Pseudo colors are applied to the $Al(OH)_3/Al_2O_3$ bi-layer film to guide the eye.

SI-3. Excluding electron beam irradiation effects on in-situ TEM observations

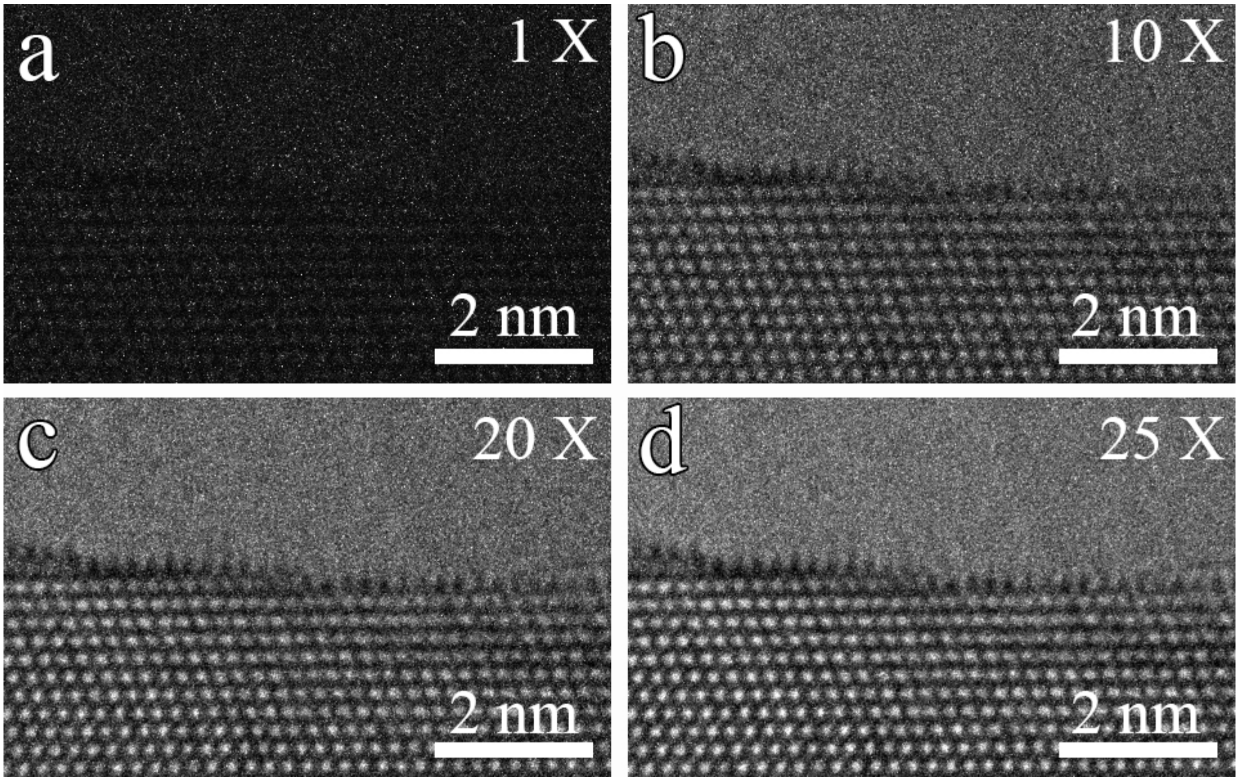

Fig. S3. Frame alignment and averaging of a series of fast "low-dose" HRTEM images. (a-d) Averaging of low-dose multiple fast HRTEM images of freshly clean Al(111) with a K2 direct electron detection camera, using the low electron flux (~ 2300 e $Å^{-2}$ s^{-1}).

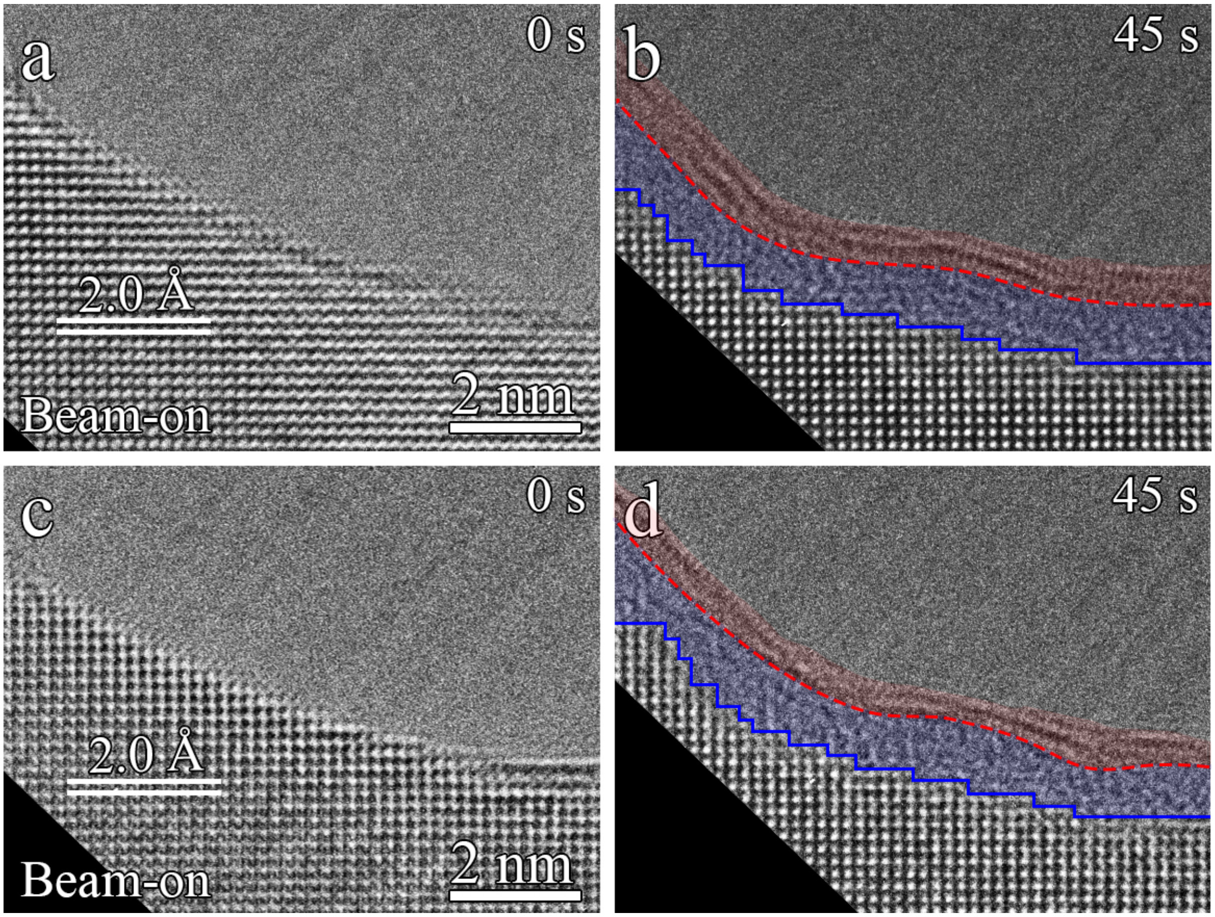

Fig. S4. "Low-dose imaging" experiments showing the $Al(OH)_3/Al_2O_3$ bi-layer film formation. (a, c) HRTEM images of freshly prepared Al surface facets viewed along the [001] zone axis. (b, d) The $Al(OH)_3/Al_2O_3$ bi-layer film formation on the Al facets during the water vapor exposure at T = 298 K and $pH_2O \approx 8.5\times10^{-5}$ Torr. The in-situ HRTEM imaging is performed with the low-dose electron flux of ~ 2300 e Å^{-2} s^{-1}. The dashed red lines and solid blue lines outline the $Al(OH)_3/Al_2O_3$ and Al_2O_3/Al interfaces, respectively. Pseudo colors are applied to the bi-layer oxides to guide the eye.

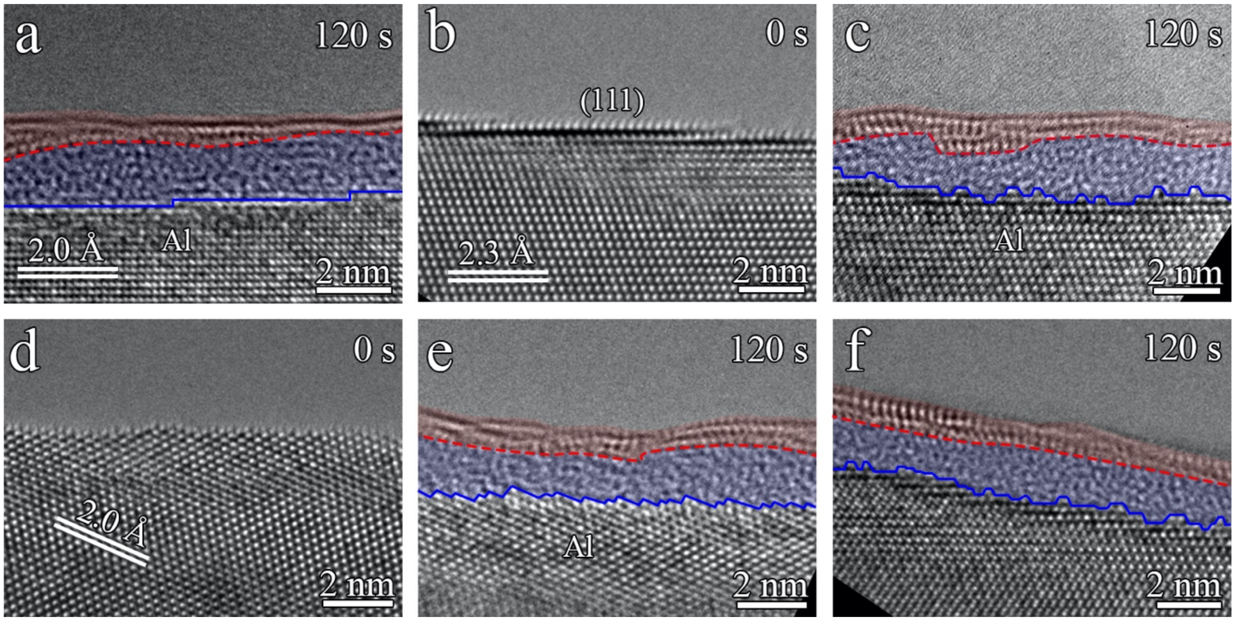

Fig. S5. "Blanked-beam" experiments showing the water vapor induced $Al(OH)_3/Al_2O_3$ bi-layer film formation on Al surfaces. (a) HRTEM image displaying the $Al(OH)_3/Al_2O_3$ bi-layer film formed "in the dark" on an Al surface region that is adjacent to the area in Fig. 3 and not subject to the e-beam irradiation during the water vapor exposure at T = 298 K and $pH_2O \approx 3.5\times10^{-4}$ Torr. (b, d) The surfaces are restored to the pristine state by sputtering off the oxide film with the condensed electron beam. (c, e, f) The electron beam is then blanked off and the pristine surfaces are subject to the water vapor exposure "in the dark" at T = 298 K and $pH_2O \approx 3.5\times10^{-4}$ Torr. The dashed red and solid blue lines mark the $Al(OH)_3/Al_2O_3$ and Al_2O_3/Al interfaces, respectively. Pseudo colors are applied to the bi-layer oxides to guide the eye.

SI-4. $Al(OH)_3/Al_2O_3$ bilayer passivating film formation on Al in deionized water

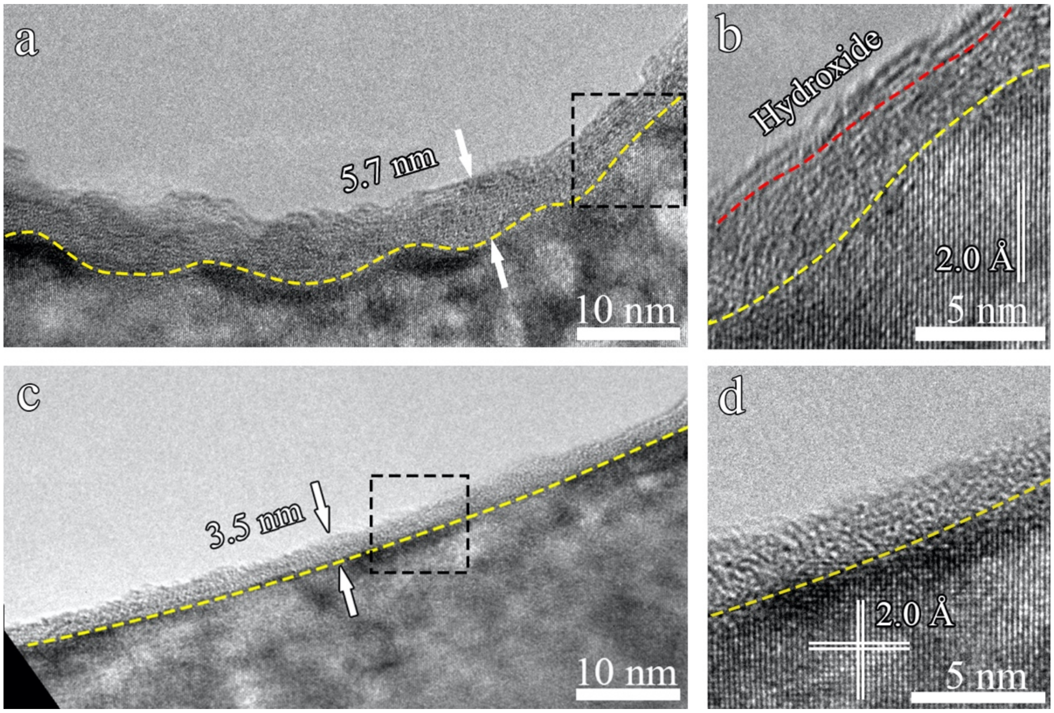

Fig. S6. Formation of the $Al(OH)_3/Al_2O_3$ bilayer film on Al by deionized water. (a) Low magnification TEM image of an FIB-prepared Al foil after being immersed in deionized water, showing the formation of a passivating oxide layer. (b) HRTEM image of the area marked by the dashed box in (a), showing the presence of a crystalline-like top layer of the Al hydroxide. (c) Low magnification TEM image of an FIB-prepared Al foil exposed to ambient air, showing the formation of a thin native oxide layer. (d) HRTEM image of the region marked by the dashed box in (c), showing the amorphous nature of the native Al oxide with the absence of the crystalline Al hydroxide. The yellow and red dashed lines mark the Al_2O_3/Al and $Al(OH)_3/Al_2O_3$ interfaces, respectively.

SI-5. Negligible effects from background gases in the TEM column on the passive oxide film formation

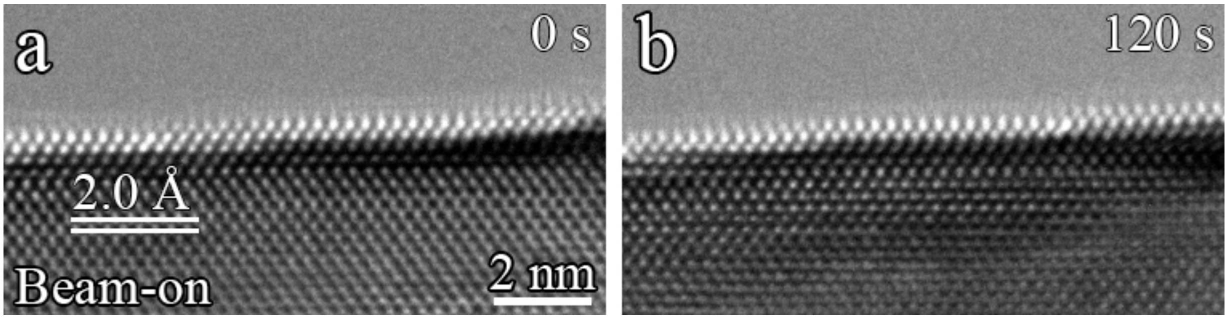

Fig. S7. Negligible effect of background gas molecules in the TEM column on the surface passivation. (a) HRTEM image illustrating a clean Al(100) surface prepared by sputtering off the native oxides. (b) The same surface area after a time elapse of 120 s under vacuum of the TEM column with a base pressure at 8×10^{-8} Torr, showing barely any oxide formation.

SI-6. XPS measurement of the passive oxide film formation on Al(111)

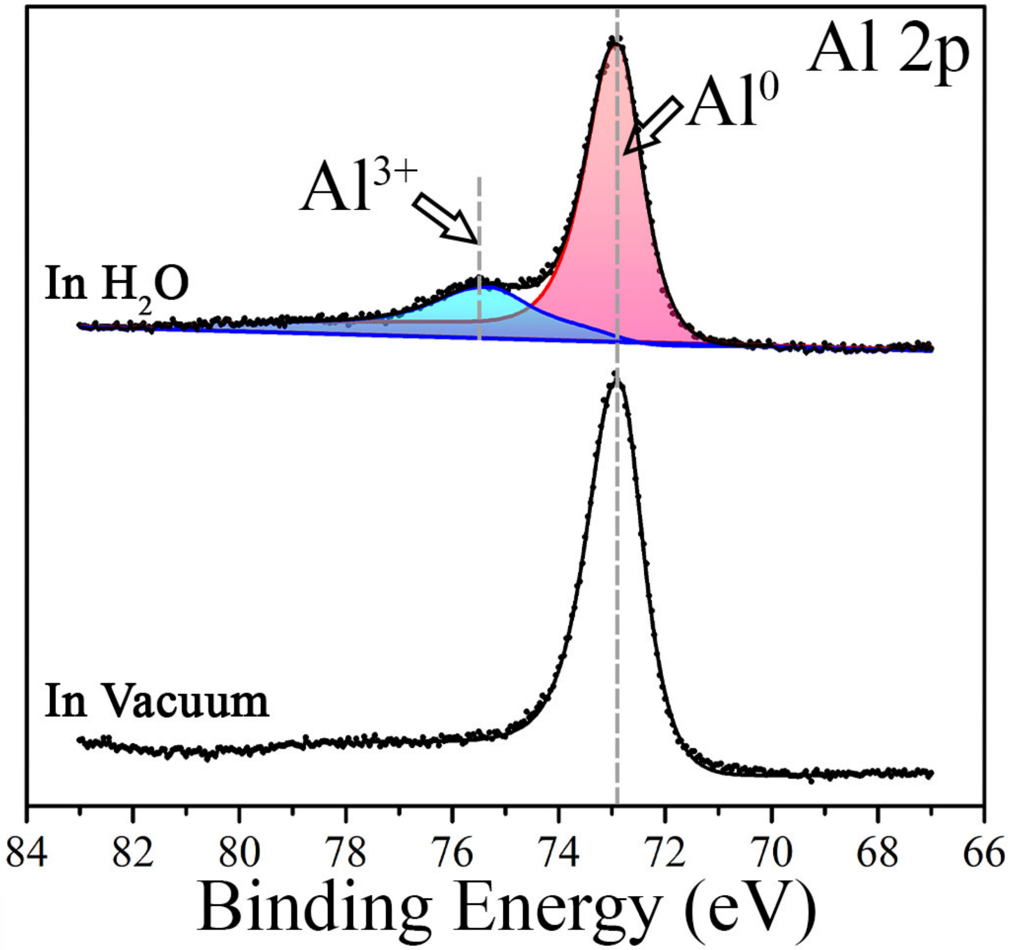

Fig. S8. XPS measurement of the passive oxide film formation on Al(111). Photoemission spectra of the Al 2p core-level region obtained from clean Al(111) (bottom panel) and after the H_2O exposure at 298 K and $pH_2O = 1\times10^{-5}$ Torr (top panel), respectively.

SI-7. *In situ* TEM observations of oxygen-induced surface passivation

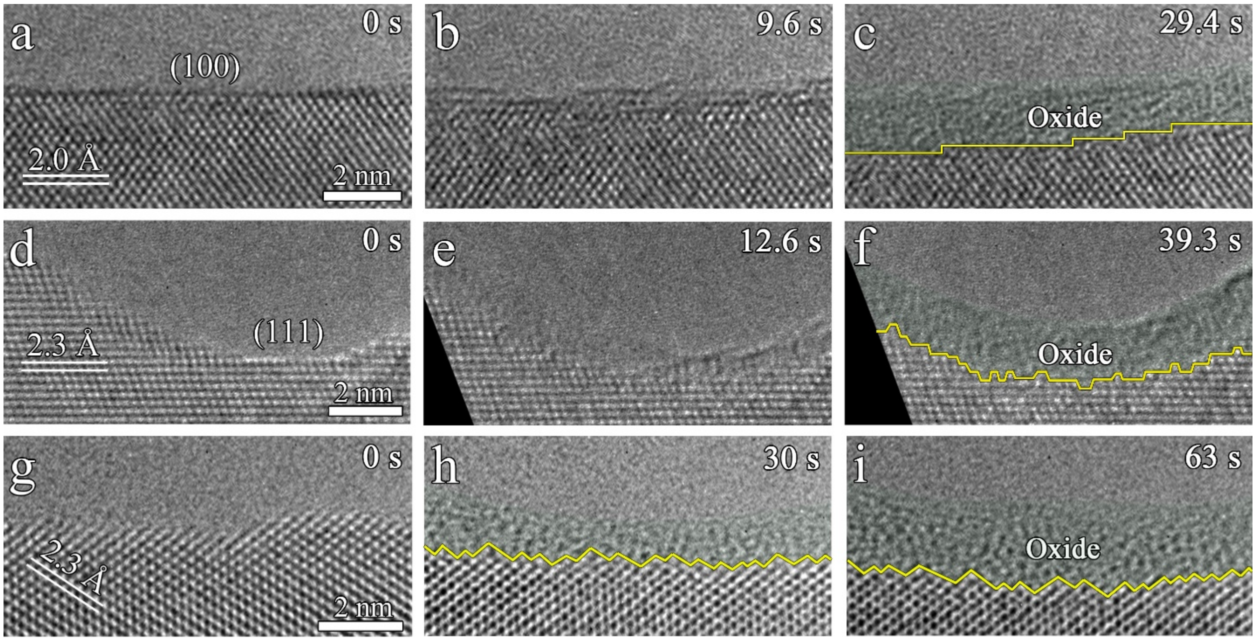

Fig. S9. **Passive oxide film formation on Al surfaces in O_2.** (a-f) Time-sequence HRTEM images showing the amorphous oxide film formation on Al(100) and Al(111) surfaces, upon the continued O_2 exposure at T = 298 K in $pO_2 \approx 8.5\times10^{-5}$ Torr. (g-i) Real time HRTEM images showing the amorphous oxide film formation on rough Al surface, upon the continued O_2 exposure at T = 298 K in $pO_2 \approx 1\times10^{-5}$ Torr. The solid yellow lines mark the Al_2O_3/Al interface. The pseudo color is applied to the oxide layer to guide the eye.

SI-8. Chemical analysis of the as-prepared Al thin foil specimens

The Al thin foils prepared using a combination of focus ion beam (FIB) and NanoMilling are examined by energy-dispersive X-ray spectroscopy (EDS). The EDS analysis is performed with Talos (FEI F200X) equipped with a four-quadrant EDS detector. Fig. S8 shows a representative EDS spectrum, in which the Mo and Cu signals are from the Mo Omniporbe grid and the Cu spacer, respectively. The absence of the signal in the Ga region confirms negligible Ga contamination from the FIB process.

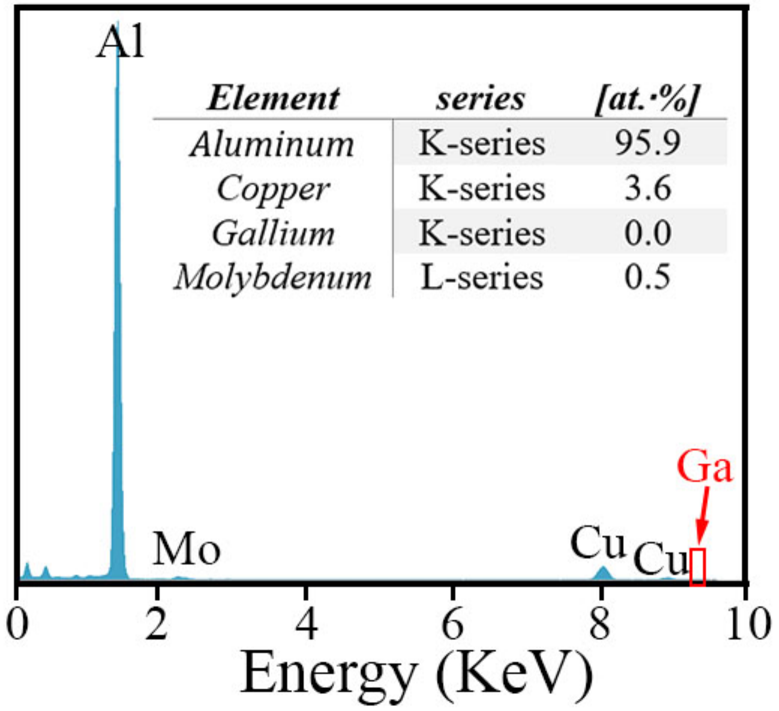

Fig. S10: **EDS spectrum collected from an FIB-prepared Al thin foil specimen.** The Mo and Cu signals are from the Mo Omniporbe grid and the Cu spacer, respectively. The absence of the peak in the Ga region (marked by the red rectangular) indicates negligible Ga contamination from the FIB milling process. The inset shows the EDS quantification the compositions.

The native oxide formed on the as-prepared Al nano-hole is characterized by TEM imaging and electron energy loss spectroscopy (EELS). Fig. S11(a) illustrates a low magnification TEM image, showing the presence of an ultrathin native oxide layer (as marked by the dashed black line) along a nano-hole in an as-prepared Al foil due to its exposure to the ambient air before loading into the TEM column. Fig. S11(b) is an HRTEM image of the native oxide, showing the amorphous nature of the Al native oxide with a thickness of ~ 4.1 nm. Fig. S11(c) shows the STEM EELS Al K-edge mapping. Fig. S11(d) corresponds to EELS spectra acquired from the native oxide and the Al substrate, respectively, as marked by the red and black dots in Fig. S11(c). Fig. S11(e) displays the STEM EELS O K-edge mapping, where the region with the higher O intensity corresponds to the native oxide formed along the hole edge. Fig. S11(f) shows the O K-edge spectra obtained from the regions marked by the red and black dots in (e).

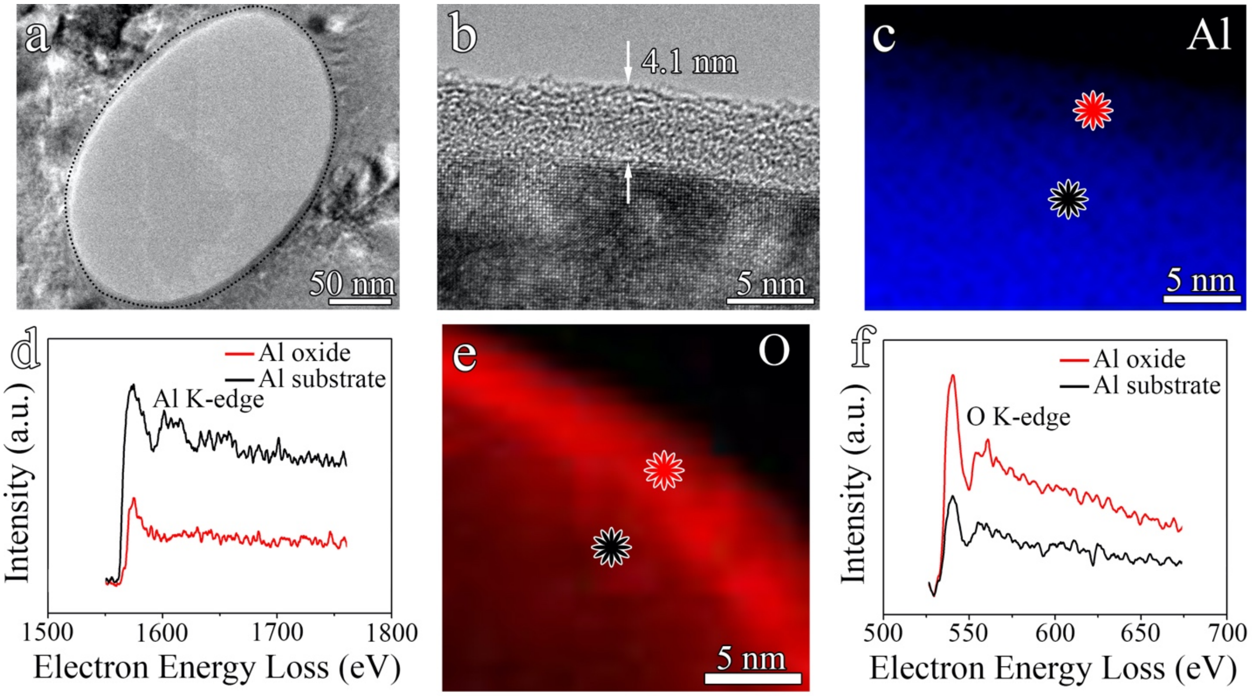

Fig. S11. Composition analysis of the native oxide formed along the edge of a nano-hole in an Al foil. (a) Low magnification TEM image of an Al foil, showing the formation of a thin native oxide layer along a nano-hole due to its exposure to ambient air. The dashed black line marks the oxide/Al interface. (b) HRTEM image displaying the amorphous nature of the native Al oxide film with a thickness of ~4 nm. (c) STEM EELS Al mapping. (d) Corresponding EELS spectra of the regions marked by the red and black dots in (c). (e) STEM EELS O mapping. (f) EELS O spectra obtained from the regions marked by the red and black dots in (e).

Caption for supplemental movies:

Supplementary Movie 1 (the movie from which Fig. 1 is extracted): *In situ* TEM movie showing the $Al(OH)_3/Al_2O_3$ bi-layer film growth on Al(111) during the water vapor exposure at T = 298 K and $pH_2O \approx 3.5\times10^{-5}$ Torr.

Supplementary Movie 2 (the movie from which Fig. 2 is extracted): *In situ* TEM movie showing the $Al(OH)_3/Al_2O_3$ bi-layer film growth on Al(100) during the water vapor exposure at T = 298 K and $pH_2O \approx 8.5\times10^{-5}$ Torr.

Supplementary Movie 3 (the movie from which Fig. 3 is extracted): *In situ* TEM movie showing the $Al(OH)_3/Al_2O_3$ bi-layer film formation in a corner region consisting of a flat (100) facet and a stepped facet during the water vapor exposure at T=298 K and $pH_2O \approx 3.5\times10^{-4}$ Torr.

Supplementary Movie 4 (the movie from which Fig. 4 is extracted): *In situ* TEM movie showing the $Al(OH)_3$ formation on an amorphous Al_2O_3 overlayer during the water vapor exposure at T=298 K and $pH_2O \approx 3.5\times10^{-4}$ Torr.

Supplementary Movies 5-7 (the movies from which Figs. 6(a-g) are extracted): ReaxFF MD simulations of the hydroxylation reaction on Al(100) during the water vapor exposure at T=298 K and $\rho H_2O = 16.5$ g/cm^3.

Supplementary Movie 8 (the movie from which Figs. 6(h-j) are extracted): ReaxFF MD simulations of the hydroxylation reaction on an amorphous Al_2O_3 overlayer during the water vapor exposure at T=298 K and $\rho H_2O = 16.5$ g/cm^3.
